# Work-related stress assessed by a text message single-item stress question

**DOI:** 10.1093/occmed/kqx111

**Published:** 2017-08-18

**Authors:** B Arapovic-Johansson, C Wåhlin, L Kwak, C Björklund, I Jensen

**Affiliations:** 1 Unit of Intervention and Implementation Research for Worker Health, Institute of Environmental Medicine, Karolinska Institute, Sweden; 2 Occupational and Environmental Medicine Center, and Department of Clinical and Experimental Medicine, Linköping University, Sweden

**Keywords:** Predictive validity, screening, single-item question, text message, work-related stress

## Abstract

**Background:**

Given the prevalence of work stress-related ill-health in the Western world, it is important to find cost-effective, easy-to-use and valid measures which can be used both in research and in practice.

**Aims:**

To examine the validity and reliability of the single-item stress question (SISQ), distributed weekly by short message service (SMS) and used for measurement of work-related stress.

**Methods:**

The convergent validity was assessed through associations between the SISQ and subscales of the Job Demand–Control–Support model, the Effort–Reward Imbalance model and scales measuring depression, exhaustion and sleep. The predictive validity was assessed using SISQ data collected through SMS. The reliability was analysed by the test–retest procedure.

**Results:**

Correlations between the SISQ and all the subscales except for job strain and esteem reward were significant, ranging from −0.186 to 0.627. The SISQ could also predict sick leave, depression and exhaustion at 12-month follow-up. The analysis on reliability revealed a satisfactory stability with a weighted kappa between 0.804 and 0.868.

**Conclusions:**

The SISQ, administered through SMS, can be used for the screening of stress levels in a working population.

## Introduction

Work-related stress is one of the factors associated with mental ill-health [1,2], and mental ill-health is one of the major causes of sickness absence in Western countries [2]. Work-related stress can be defined as a negative reaction to work conditions accompanied by high levels of distress and arousal [3]. Prolonged distress and arousal can lead to psychological strain and illness, both physical and mental [2,4].

In Sweden, where the present study was conducted, almost 15% of women and 8% of men in the working population report work-related stress [5]. Furthermore, in the health care sector, >60% of midwives, nurses and health care specialists feel that workload is excessive [6]. In view of this, measures of stress should be given high priority. These measures need to be easy to use and acceptable to employers and employees. A single-item question, administered by text message (short message service (SMS)), is one possible solution. Text messages, as a method for data collection, have high response rates [7]. The combination of a validated single-item measure and SMS makes it possible to continuously monitor levels of stress and take early action. Littman *et al*. [8] validated two single-item measures of stress for use in large epidemiological studies. However, they are less appropriate for predictive purposes because they capture perceived stress in the previous year rather than the ongoing experience of stress-related symptoms.

The single-item stress question (SISQ) [9] is widely used [10,11], but its predictive validity and reliability have not been assessed previously. Nor has it been administered through SMS. The SISQ captures subjective experience of stress, and can be seen as a global indicator of stress, dependent on multiple causal sources (working conditions, individual factors, life circumstances, etc.). Theoretical predictions are essential for statements about validity [12]. If SISQ is to be a valid measure of the experience of stress at work, it should be associated with well-known work-related stressors and other constructs within work-stress research, such as exhaustion. The Job Demand–Control–Support (JDC-S) model [4] and the Effort–Reward Imbalance (ERI) [13] model are two well-known models of work-related stress. Job strain is a measure of the balance between job demand and job control and would be expected to have a positive association with SISQ. Social support (supervisor and co-worker) has a buffering effect on stress [14], thus implying negative associations with SISQ. The over-commitment subscale of the ERI model captures individual differences in patterns of excessive work-related commitment, and the correlation with the SISQ would be expected to be positive. The relationship between stress, sleep difficulties, depression and exhaustion is well known [1,2].

Another important aspect of validity is predictive validity [15]. Sickness absence, depression and exhaustion have well-researched associations with work-related stress. The test–retest reliability of the SISQ is a prerequisite for its validity. The aim of this study was to examine the convergent validity, the predictive validity and the test–retest reliability of the SISQ when distributed by SMS.

## Methods

The validation was carried out as part of a randomized controlled trial (for more information, see ClinicalTrials.gov, ID: NCT02694211). Participants were employed at three team-based primary health care facilities. Primary health care in Sweden is responsible for treating diseases and injuries when hospitalization is not necessary. Primary health care physicians are specialists in general practice. Other staff categories include nurses, midwives, physiotherapists, counsellors, biomedical technicians, etc.

**Figure 1.**
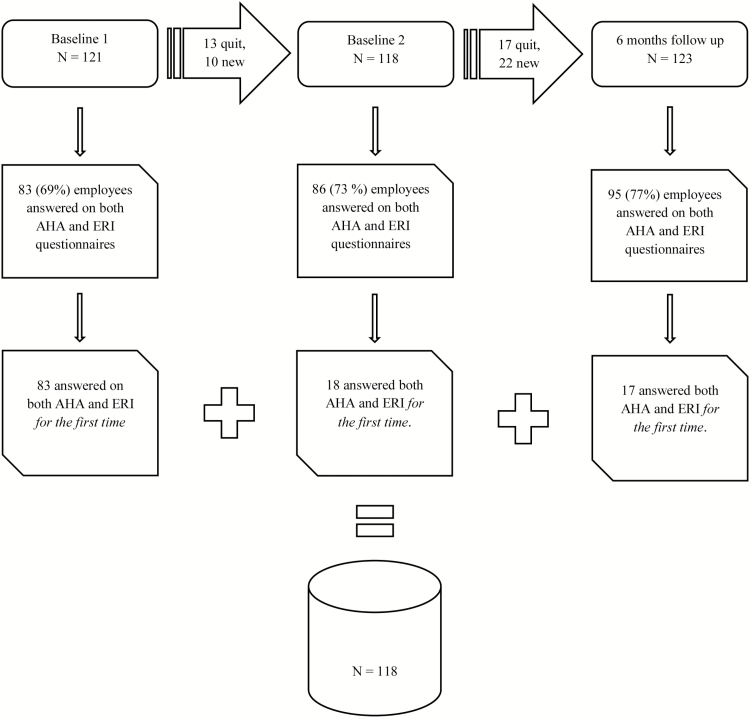
The flow of participants in the convergent validation study*. *One of the employees moved from one unit to another. Therefore, the total number of employees employed at some point in time is 121 + 10 + 22 − 1= 152. The response rate is 118/152 = 78%.

A comprehensive questionnaire was used for two baseline measurements, and at 6- and 12-month follow-ups. Text messages with the SISQ were sent weekly for 12 weeks at the beginning of the intervention. The data from the two baseline measurements and the 6-month follow-up were used to examine convergent validity. Data were collected from employees who answered the SISQ and the questionnaires for the first time (Figure 1).

**Figure 2.**
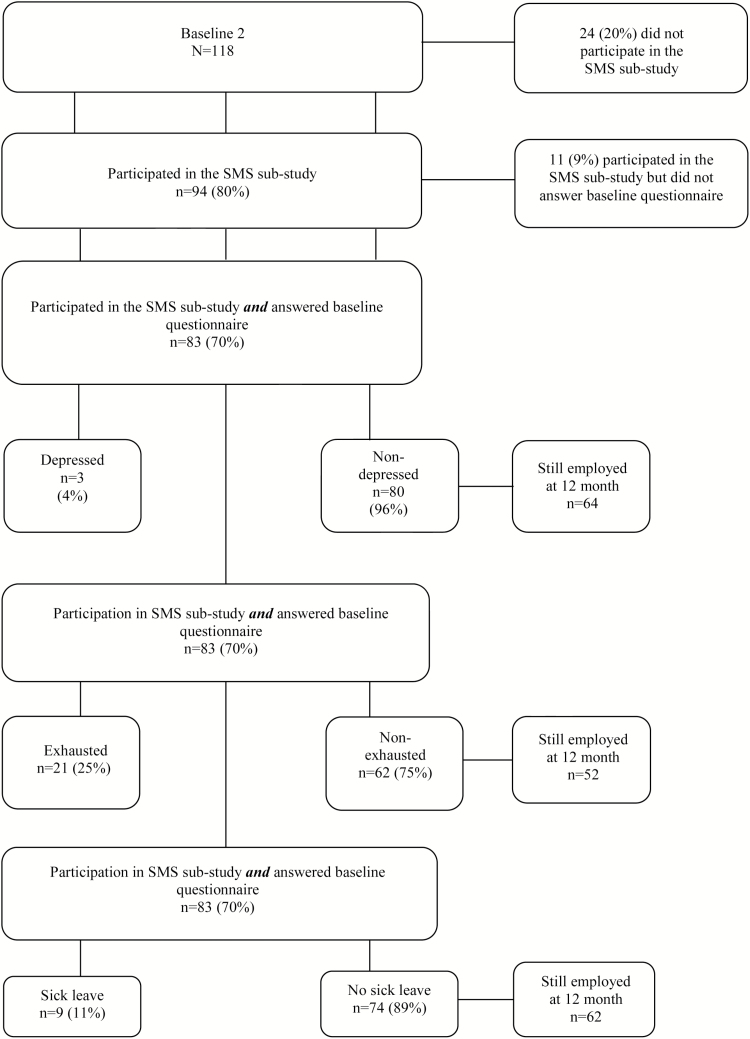
Flow chart predictive validity.

To examine the predictive validity, we included those employees who answered the weekly SMS as well as the 12-month follow-up questionnaires (Figure 2). The variables were sickness absence, depression and exhaustion. Only employees who took no sick leave or had no signs of depression or exhaustion at baseline were selected.

The Swedish version of the SISQ was used to measure the experience of stress, namely: ‘Stress means a state in which a person feels tense, restless, nervous or anxious or is unable to sleep at night because his/her mind is troub led all the time. Do you feel this kind of stress these days?’ [9,10]. The responses were recorded on a 5-point Likert scale. The questionnaire used to measure the constructs in the JDC-S model is a compilation of validated instruments developed in the AHA study [16] to assess the psychosocial work environment, health and lifestyle. The sections concerning the psychosocial work environment are based on QPS-Nordic [10]. The job demand subscale has seven questions; job control, eight questions; social support from leadership, three questions; and social support from co-workers, two questions. The responses were measured on a 5-point Likert scale. Job strain is the relationship between high job demand and low job control. The Swedish validated version of the ERI questionnaire [17] was used to measure the constructs of the ERI model: six questions about effort, 11 about reward and six about over-commitment. Answers were given on a 4-point Likert scale. Effort–reward imbalance is a ratio of effort and reward subscales.

Overall sleep quality was measured by a single question from the Swedish WOLF study [18]: ‘How would you assess your overall sleep quality?’ The response was recorded on a 5-point scale varying from ‘very good’ (1) to ‘very bad’ (5). Sleep problems due to thinking about work were measured by the question from the Swedish ‘Schools in Development’ project [19]: ‘Does it ever happen that you find it difficult to sleep, that you’re lying awake thinking about work?’

Exhaustion was measured using the validated Swedish version of the Oldenburg Burnout Inventory (OLBI) [20], while depression was measured with seven questions from the validated Swedish version of the Hospital Anxiety and Depression Scale (HADS) [21]. Sickness absence was measured with the question: ‘How many of the past seven working days have you missed because of sick leave?’ The question is adapted from a question used in work productivity research [22].

Internal consistency of subscales used in validation of SISQ was assessed by computing Cronbach’s alpha. Convergent validity was analysed using Spearman’s rho, while the General Linear Model (GLM; univariate) was used to analyse predictive validity. The aim of the GLM analysis was not to build a model to explain sick leave, depression and exhaustion, but rather to examine whether the SISQ by itself, administered by SMS, could predict sick leave, depression and exhaustion at 12-month follow-up. The main analysis was therefore without factors and covariates. However, in order to further examine the SISQ’s predictive power, we followed up the main analysis with an analysis in which depression (known to be associated with sick leave) was included as a covariate for sick leave as an outcome, and job strain was included as a covariate when depression and exhaustion were outcomes. The primary independent variables of interest were the mean values for the SISQ for weeks 1–4, 1–8 and 1–12. Given our results, during the analysis, we decided to repeat the intervals (examine the predictive validity of the mean values of all pairs of weeks, i.e. week 1–2, 2–3, 3–4, etc.).

A test–retest procedure was used to assess the reliability of the SISQ. Study participants were a convenience sample. The inclusion criteria were: being older than 18 years, being gainfully employed (not on sick leave), having access to a mobile phone and agreeing to participate in the study. Participants were told they would receive four questions by SMS message, within a 24-h period. Question 1 on Tuesday was: ‘Does this day differ in any way from your “typical day?” (answer Y for Yes and N for No).’ After receiving the answer, the automated system would send Question 2 (the stress question). On Wednesday, the first question was the stress question, followed by: ‘Does this day differ in any significant/noticeable way from yesterday?’ The aim of asking these two supplementary questions was to compare the reliability of the SISQ in the group where no change between days was reported with the reliability of SISQ for the whole group of participants. The SMS-Track Questionnaire software system [23] was used to distribute the messages and collect the answers. It has previously been tested in research settings [24]. Data about sex and age were collected by e-mail and phone.

Weighted kappa was used to analyse the test–retest reliability and was calculated both with and without selection. Without selection means that no notice was paid to whether participants answered yes or no to the supplementary questions. With selection means that only subjects who answered no to both supplementary questions were used in the analyses. IBM SPSS statistics version 22 was used for all analysis except weighted kappa, for which we wrote a program according to the formula for weighted kappa (squared weights) [25].

The Stockholm Regional Ethical Review Board approved this project (2012/2200-31/5).

## Results

The sample in the validation study (see Figure 1) consisted of 118 subjects (19 males and 99 females). Table 1 displays descriptive data for the validation study population, and Table 2 displays descriptive data for the SISQ and the validation subscales.

**Table 1.** Descriptive background data for the population in the validation study (total and divided by gender)

	Total (*n* = 118)	Female (*n* = 99)	Male (*n* = 19)
Age, years, mean (SD)	44.6 (11.7)	44.4 (11.8)	45.3 (11.7)
Work hours/week, mean (SD)	36.8 (6.7)	36 (6.5)	39 (7.0)
Overtime hours/months, mean (SD)	7.8 (27.2)	5.2 (16.5)	21.2 (55.8)
Employed >10 years, *n* (%)	22 (19)	20 (20)	2 (11)
Immigrant, *n* (%)	12 (10)	12 (12)	0
Education level, *n* (%)			
Primary school	1 (1)	1 (1)	0
Secondary school	17 (14)	17 (17)	0
University education	95 (81)	79 (80)	16 (84)
Higher academic education	5 (4)	2 (2)	3 (16)
Profession, *n* (%)			
Physician	18 (15)	9 (9)	9 (47)
Nurse	37 (31)	35 (35)	2 (11)
Physical therapist	18 (16)	15 (15)	4 (21)
Medical secretary	14 (11)	13 (13)	0
Counsellor	5 (4)	3 (3)	2 (11)
Midwife	8 (7)	8 (8)	0
Laboratory technician	8 (7)	8 (8)	0
Assistant nurse	7 (6)	7 (7)	0
Manager	2 (2)	0	2 (10)
Dietitian	1 (1)	1 (1)	0

**Table 2.** Median, range, minimum, maximum and number of items for the SISQ, and relevant validation subscales used as reference measures in the validation (Cronbach’s alpha for subscales)

Subscale	Median	Range	Min/max	Cronbach’s alpha	No. of items
SISQ (1–5)	3	4	1/5	–	1
Job demand (1–5)	3.5	2.86	2.0/4.9	0.807	7
Job control (1–5)	2.75	3.38	1.0/4.4	0.806	8
Co-worker support (1–5)	4.5	3.5	1.5/5.0	0.894	2
Leadership support (1–5)	4	4	1/5	0.791	3
Effort (6–24)	13	13	7/20	0.715	5
Reward (11–44)	35	28	15/43	0.782	11
Over-commitment (6–24)	13	18	6/24	0.862	6
Global sleep quality (1–5)	2	4	1/5	–	1
Sleep difficulties^a^ (1–5)	2	3	1/4	–	1
Depression (0–21)	9	16	7/23	0.863	7
Exhaustion (8–32)	19	18	10/28	0.815	8

^a^Sleep difficulties due to thinking about work.

Table 3 displays the correlations between the SISQ and the subscales for the total study population. As hypothesized, there was a significant positive association between SISQ and job demand, effort, over-commitment, exhaustion and depression. The results also demonstrate significant negative associations between SISQ and social support (both supervisor and co-worker support), as well as SISQ and job control and reward. The positive association between SISQ and job strain was non-significant.

**Table 3.** Correlations (Spearman’s rho) between the SISQ and validation subscales for the total study population

Validation of subscale	SISQ
	Total (*n* = 118)
Job strain	0.182
Job demand	0.357**
Job control	−0.218*
Co-worker support	−0.299**
Leadership support	−0.199*
Effort–reward ratio	0.467**
Effort	0.330**
Reward	−0.347**
Over-commitment	0.627**
Global sleep quality	−0.321**
Sleep difficulties^a^	0.566**
Depression	0.456**
Exhaustion	0.580**

^a^Sleep difficulties due to thinking about work.

*P < 0.05 level (two-tailed). **P < 0.01 level (two-tailed).

As shown in Table 4, the results show that the SISQ significantly predicted sickness absence at 12-month follow-up. The significant results remained for the SISQ mean for weeks 1–8 (*B* = 0.369, SE = 0.167, 95% CI 0.034 to 0.704, *P* < 0.05) and weeks 1–12 (*B* = 0.409, SE = 0.169, 95% CI 0.070 to 0.748, *P* < 0.05), even when depression at baseline was included in the model as a covariate (data not presented in the table).

**Table 4.** Predictive validity of the SISQ

Dependent variable	Predictor variable: SISQ (mean value)	*B*	SE	*t*	*P*	95% confidence interval	
						Lower bound	Upper bound
Sick leave^a^	(No covariates)						
	Week 1–2	0.357	0.151	2.299	<0.05	0.045	0.648
	Week 2–3	0.302	0.131	2.308	<0.05	0.040	0.563
	Week 3–4	0.281	0.135	2.085	<0.05	0.011	0.550
	Week 4–5^d^	0.285	0.145	1.968	NS	−0.005	0.575
	Week 5–6^d^	0.279	0.148	1.887	NS	−0.017	0.576
	Week 6–7	0.293	0.162	1.815	NS	−0.030	0.617
	Week 7–8	0.452	0.161	2.808	<0.01	0.130	0.775
	Week 8–9	0.392	0.148	2.649	<0.05	0.096	0.688
	Week 9–10	0.370	0.149	2.487	<0.05	0.072	0.668
	Week 10–11	0.372	0.146	2.548	<0.05	0.080	0.665
	Week 11–12	0.369	0.147	2.512	<0.05	0.750	0.663
	Week 1–4	0.355	0.151	2.354	<0.05	0.053	0.657
	Week 1–8	0.413	0.162	2.557	<0.05	0.090	0.736
	Week 1–12	0.451	0.165	2.737	<0.01	0.121	0.780
Depression^b^	(No covariates)						
	Week 1–4	1.041	0.339	3.067	<0.01	0.362	1.719
	Week 1–8	0.874	0.376	2.325	<0.05	0.123	1.626
	Week 1–12	0.997	0.384	2.597	<0.05	0.230	1.764
	(Job strain a covariate)						
	Week 1–4	1.016	0.366	2.772	<0.01	0.283	1.748
	Week 1–8	0.809	0.403	2.008	<0.05	0.004	1.614
	Week 1–12	0.939	0.409	2.299	<0.05	0.122	1.756
Exhaustion^c^	(No covariates)						
	Week 1–4	1.753	0.495	3.542	<0.001	0.759	2.748
	Week 1–8	1.796	0.540	3.328	<0.01	0.712	2.880
	Week 1–12	1.432	0.573	2.499	<0.05	0.281	2.583
	(Job strain a covariate)						
	Week 1–4	1.652	0.518	3.192	<0.01	0.612	2.692
	Week 1–8	1.680	0.565	2.971	<0.01	0.544	2.816
	Week 1–12	1.288	0.591	2.177	<0.05	0.099	2.476

GLM (univariate). Dependent variables: sick leave, depression and exhaustion at 12-month follow-up. Only employees without sick leave, depression and exhaustion at the baseline measurement are included. NS, non-significant.

^a^Individuals without job strain did not have any sick leave.

^b^Measured by HADS (Lisspers *et al*. [21]; Zigmond and Snaith [29]).

^c^Measured by OLBI (Demerouti *et al*. [30]; Peterson [20]).

^d^Week 5 is a fall break.

Table 4 also displays the results of the regression analysis with depression and exhaustion as dependent variables. The SISQ was a significant predictor of depression even when job strain at the baseline was included as a covariate. When measured at weeks 1–4, 1–8 and 1–12, the SISQ was also a significant predictor of exhaustion at the 12-month follow-up, both alone and with job strain as a covariate in the model.

Of 108 subjects (27 males, 81 females) who participated in the test–retest procedure, only subjects who answered on both days were included in the analysis (*n* = 99, 92%, 24 males, 75 females). The ages ranged between 25 and 67 years; the mean age was 48.3 years (SD 10.23). Sixty-seven per cent (66 individuals) answered no to the question: ‘Does this day somehow differ from your “typical day?” ’ On day 2, 74% (77) answered no to the question: ‘Does this day differ from yesterday in any significant/noticeable way?’ Reliability analyses were made both without selection (irrespective of whether answers to these questions were yes or no) and with selection (only subjects who answered no to both questions). The analyses of test–retest reliability revealed a satisfactory stability [26]: weighted kappa without selection was 0.804 (*n* = 99) and with selection 0.868 (*n* = 52).

## Discussion

Our study found that the SISQ, distributed by weekly SMS, could predict future sickness absence, depression and exhaustion. Furthermore, it was associated with a number of validated subscales that measure important constructs for work-related stress, as would be expected by theories within the field. The estimation of reliability showed that it was a stable enough measure for use in data collection by means of SMS.

The results are promising. The symptoms which the SISQ seems to capture successfully are early signs of a long process that can end in exhaustion and sickness absence. The ability to distribute the SISQ by SMS over 2–4 weeks and to be able to predict who is at risk of sickness absence or exhaustion would be a practical tool. It could be a starting point for closer examination and actions to prevent chronicity.

One limitation of this study is that SISQ is constructed without explicit reference to work. It could be argued that the question captures overall stress rather than work-related stress, which could then be said to compromise its validity as a work-related measure of stress. However, we argue that it is an individual’s total experience of stress that will affect their performance at work, sickness absence, help-seeking behaviour, etc. Furthermore, the question was asked in a workplace setting and it also demonstrates the highest correlation with the work-related scales. The SISQ demonstrated the strongest association with the ERI subscale of over-commitment. Four out of six questions about over-commitment refer to work and work-related problems. The SISQ also shows a much higher correlation with the question about difficulty in sleeping because of thinking about work than with general sleep quality, which could be another indicator of its relevance in a work-related context. However, this item could be sensitive to other factors not related to the psychosocial work environment and this should be taken into consideration when using the item.

Overall, the associations between the SISQ and comparison subscales were consistent with theoretical predictions. The SISQ had a stronger association with effort–reward imbalance than with job strain. According to some studies, the ERI model may have more power to explain the experience of stress in service occupations, such as the health care professionals used in our study [27]. The study shows that, in this population, an employee’s perception of being under stress is correlated with his/her perception of job demand and experience of effort–reward imbalance. The results of validation can be generalized to employees in primary health care settings with a predominance of highly educated middle-aged women. Additional studies are needed if we are to be able to draw conclusions about the male population or professions other than the health care sector.

The research community seems to be divided over the reliability of single-item questions [28]. We argue that stress (as defined in this paper), even though a complex and fluctuating condition, can nevertheless be meaningfully measured by the SISQ, and that the SISQ’s reliability can be estimated by adapting methods (as here) and/or by combining different methods of assessment [28]. Its test–retest reliability is high when measured in a time frame more suitable for fluctuating conditions. The test–retest procedure is usually seen as appropriate only for measures of relatively stable concepts, for example personality traits [15]. Assessing test–retest reliability for fluctuating conditions is seen as having little sense [15]. We argue that the definition and choice of time interval between the test and retest is a crucial factor when considering the reliability of measures for fluctuating conditions. The ‘time increases, correlation decreases’ principle is still valid, but the interval between the test and the retest should be shorter than for more stable constructs. Exactly how short depends on the construct, and needs to be explored empirically for every fluctuating condition. For the SISQ, if administered on two consecutive days, the test–retest reliability should be fairly high at group level if the population is not a psychiatric population or one with serious memory impairment. A correlation of 1.0 would mean that there is no change in experience of stress level from one day to another at a group level. That might indicate that the question is not at all sensitive to possible changes. However, if the reliability is too low when measured at shorter intervals (here two consecutive days), it would indicate that it is too unstable. With the question ‘Does this day differ from yesterday in any significant/noticeable way?’ we tried to filter out two groups of subjects, and indeed the reliability (stability in answers) is somewhat higher for the group that answered no to this question, which also supports the reliability of the SISQ.

Internal reliability is a concept developed and used for multiple-item questionnaires. It is not surprising if a tool developed for one field cannot easily be used in a different field, such as single-item questions. This does not necessarily mean that we should abandon single items, not even for fluctuating states. Rather we need to put effort into finding new ways to assess their suitability for research and practice. To assume that the reliability of a single-item question for a fluctuating condition can never be assessed, may cost us relevant, acceptable and cost-effective tools.

The validation of measures is an ongoing process in research, and convergent and predictive validity are important aspects of that process [31]. This validation of the SISQ shows that it is a promising tool in stress-prevention research and practice. Given the prevalence of work-stress related ill-health in the Western world, it seems important to find cost-effect ive, easy-to-use and valid measures which can be used both in research and in practice. A combination of the SISQ, SMS and wearables technology could highly improve data collection, both response rates and accuracy. SISQ could be used in organizations as a highly practical and sustainable tool for the regular screening of stress levels at group/organizational level. It is a simple and feasible method for the early identification of individuals at risk of sick leave and exhaustion.

**Key points**

- Given the prevalence of work stress-related illness in the Western world, it is important to find cost-effective, easy-to-use and valid measures which can be used both in research and in practice.
- The combination of a validated single-item stress question and new technology might be used as a practical and reliable tool for regular screening of stress levels at organizational level.
- In this study, the single-item stress question administered by weekly short message service was a valid, reliable and feasible method for the early identification of individuals at risk of sickness absence and exhaustion.

## Conflicts of interest

None declared.
